# Unlocking the therapeutic potential of *Nigella sativa* extract: phytochemical analysis and revealing antimicrobial and antioxidant marvels

**DOI:** 10.1186/s12906-024-04470-w

**Published:** 2024-07-12

**Authors:** Anees Ur Rahman, Abdullah Abdullah, Shah Faisal, Basem Mansour, Galal Yahya

**Affiliations:** 1 Department of Health and Biological Science, Abasyn University, Peshawar, 25000 Pakistan; 2 Department of Physical Chemistry and Technology of Polymers, Silesian University of Technology, M. Strzody 9, Gliwice, 44-100 Poland; 3 Joint Doctoral School, Silesian University of Technology, Akademicka 2A, Gliwice, Poland; 4 Center for Health Research, Guangzhou Institute of Biomedicine and Health, Chinese Academy of Sciences, Guangzhou, 510530 China; 5 University of Chinese Academy of Sciences, Beijing, 100049 China; 6 Institute of Biotechnology and Microbiology, Bacha Khan University, Charsadda, 24460 Pakistan; 7 Department of Pharmaceutical Chemistry, Faculty of Pharmacy, Delta University for Science and Technology, Gamasa, 11152 Egypt; 8 Department of pharmaceutical chemistry, Kut University College, Al Kut, Wasit, 52001 Iraq; 9 Department of Microbiology and Immunology, Faculty of Pharmacy, Zagazig University, Al Sharqia, 44519 Egypt

**Keywords:** Black seed, Antimicrobial, Synergism, Traditional medicine, Antioxidant, Antiparasitic

## Abstract

The growing global threat of antimicrobial resistance endangers both human and animal life, necessitating the urgent discovery of novel antimicrobial solutions. Medicinal plants hold promise as sources of potential antimicrobial compounds. In this study, we investigated the phytochemical constituents and microbicidal capabilities of the ethanolic extract from *Nigella sativa* (black seed). Gas chromatography analysis (GC) identified 11 compounds, among them thymoquinone, and thymol, contributing to antimicrobial and antioxidant properties. Antimicrobial assays demonstrated notable inhibition zones against broad spectra of bacteria, including *Pseudomonas aeruginosa*, *Escherichia coli*, *Salmonella typhi*, *Staphylococcus aureus*, *Enterobacter*, and *Bacillus subtilis*, along with potent antifungal activity against *Aspergillus niger*, *Penicillium*, and *Candida albicans*. Notably, when combined with antibiotics, the extract displayed exceptional synergistic antimicrobial efficacy. The black seed extract demonstrated membrane-damaging activity and disrupted virulence factors that protect microbes from antimicrobial agents, including the formation of bacterial biofilm and protease secretion. Thymoquinone, the primary active constituent of the extract, exhibited similar antimicrobial and ant virulence properties. In silico analysis targeting key regulators of quorum sensing and biofilm formation in *P. aeruginosa*, such as *RhlG*, *LasR*, and *PqsR*, showed a remarkable affinity of thymol and thymoquinone for these targets. Moreover, the *N. sativa* extract exhibited dose-dependent cytotoxicity against both the promastigote and amastigote forms of *Leishmania tropica* parasites, hinting at potential antiparasitic activity. In addition to its antimicrobial properties, the extract displayed potential antioxidant activity at a concentration of 400 μg/mL.

## Introduction

The escalating threat of antimicrobial resistance poses a rapidly growing global peril, imperiling both human and animal lives [1, 2]. Numerous microbes and parasites have developed resistance to a majority of antimicrobial agents, intensifying the urgency and complexity of finding or developing new ones [3]. Medicinal plants emerge as a valuable source for potential antimicrobial compounds [4–9]. Among these botanical sources, *Nigella sativa* (black seed), an herbaceous plant esteemed for its culinary applications [10], particularly in the baking and food industries, has also been harnessed for its traditional medicinal attributes in various diseases, including bacterial infections, dyslipidemia, diarrhea, inflammation, diabetes, and asthma [11–13]. Notably, black seed contains a diverse array of phytochemicals, essential oils, alkaloids, saponins, polyphenols, and proteins contributing to the aforementioned properties [14, 15]. The bioactive constituents in black seed exhibit potent properties, positioning it as a robust natural remedy for preventing, improving, and managing diseases [16]. It underscores its potential as a potent antitumor, antiallergic, antioxidant, antiestrogenic, and antibacterial agent. *N. sativa* seeds encompass over 100 different compounds, with thymoquinone being a significant constituent. The medicinal properties of thymoquinone strongly support the therapeutic potential of *N. sativa* [17].

The compounds found in *N. sativa* extracts permeate microbial cells, interacting with proteins and DNA, exerting a detrimental effect on bacterial cell viability [18]. Extensive research on plant extracts has demonstrated their noteworthy influence in pharmaceutical and biological fields, with their primary focus on reducing the permeability of bacterial cell walls, subsequently limiting their metabolic activity [18, 19].

The substantial biomedical properties of black seed have spurred our interest in investigating the use of the extract in conjunction with antibiotics to enhance their effectiveness against antibiotic-resistant pathogens. Our study involved the preparation of an ethanolic extract of *N. sativa* seeds, which was subsequently characterized using Fourier transform infrared spectroscopy (FTIR) and gas chromatography (GC) for phytochemical elucidation. Additionally, we conducted a comprehensive investigation into the antimicrobial, ant virulence, antioxidant, and antiparasitic properties of the *N. sativa* extract.

## Methods and materials

### Preparation of N. sativa seed extract

**Fig. 1:**
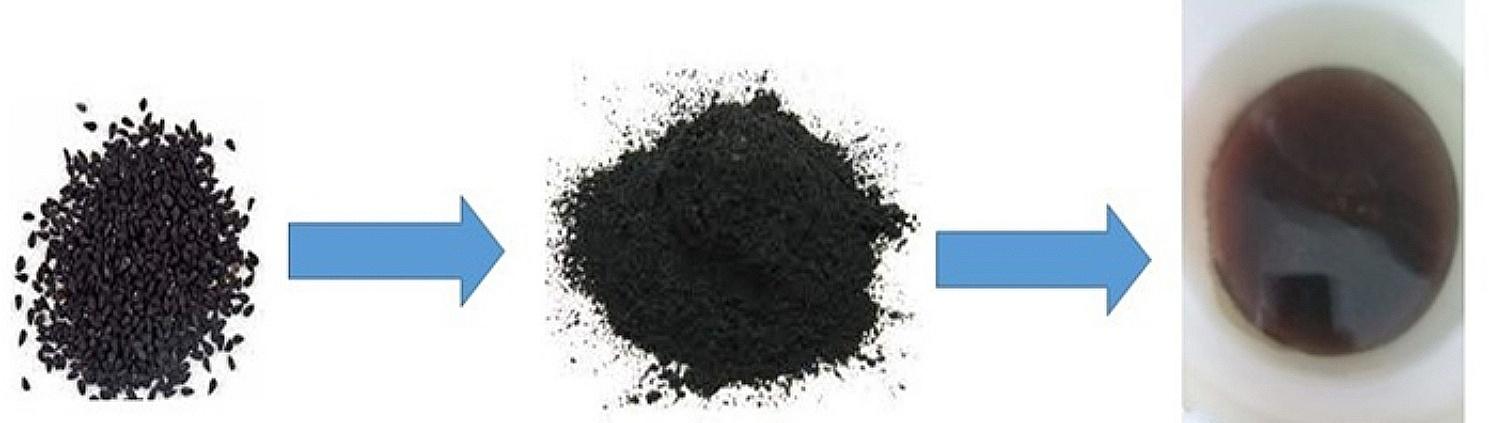
Preparation of *N. sativa* seed extract. The seeds were ground and mixed with ethanol for extraction of biocomponents

The seeds of *N. sativa* were obtained from a local market in the region of Peshawar, Khyber Pakhtunkhwa, Pakistan. These seeds are sold under various brands and names. The extract was prepared by using Solvent Extraction Method. The seeds were rinsed with water to remove any dust or other particles, dried at room temperature to eliminate any leftover moisture/dampness, and ground into powder form using an electric grinder. *N. sativa* seed powder (160.0 g) was added to 1000 mL of ethanol in a flask. The mixture was then kept at 37 °C for 48 h for maximum extraction. The extract was then filtered *via* Whatman filter paper No. 1. The filtrate was then evaporated using a rotary evaporator at 45 °C, and the crude ethanolic extract was obtained as shown in Fig. 1 [20, 21].

### FTIR analysis and GC analysis

The purified *N. sativa* ethanolic extract was analyzed by using a Perkin Elmer FTIR spectrometer (II), and measurements were taken in the wavelength range of 400 to 4000 cm^− 1^. Gas chromatography (7890B/5977B, Agilent Technologies USA) was employed for the detection of bioactive compounds in the ethanolic extract of *N. sativa* according to the detailed protocol [22]. The compounds in the extract were identified by comparing their mass spectra and retention indices to those found in the National Institute of Standards and Technology (NIST) library.

### Antibacterial activity

The antibacterial potential of the *N. sativa* seed extract was evaluated against different gram-positive (*Staph. aureus* and *B. subtilis*) and gram-negative (*P. aeruginosa*, *E. coli*, *Enterobacter*, and *S. typhi*) bacteria by using the well diffusion method [23, 24]. Nutrient agar, TSB, and skim agar media were prepared according to standard protocols and then sterilized in an autoclave at 121 °C for 45 min. After sterilization, the medium was cooled and poured into plates in a biosafety cabinet. The plates were then kept for 24 h. for the sterility check. After a sterility check, a bacterial lawn was made on nutrient agar using sterile cotton swab (Merck, Germany) plates. The wells were made in the media with the help of a sterile well borer, and then extract from the stock solution was poured into the wells. The plates were then incubated in an incubator at 37 °C for 24 h. Finally, the zone of inhibition was measured in millimeters (mm) according to [25, 26]. Ciprofloxacin was used as a positive control, while distilled water was used as a negative control.

### Minimal inhibitory concentration (MIC) and membrane damage assay

The MIC of the *N. sativa* extracts was measured in 96-well plates, with each row containing 12 wells. Nutrient broth and potato dextrose broth were prepared and sterilized using an autoclave. After cooling to room temperature, the media were poured into each well of the plate, nutrient broth for bacteria, and potato dextrose broth for fungi. In each row, the first well was used as a negative control, while in wells 2–11, 0.3 mL of extra medium containing culture was added. Antibiotics were added to well No. 12, which served as a control. The plates were then incubated for 24 h at 37 °C for bacteria and 28 °C for fungi. The OD 600 nm was measured after 24 h using a spectrophotometer [27–30].

The impact of the prepared *N. sativa* seed extract on the structural integrity of bacterial cell membranes was examined. Upon interaction of the *N. sativa* seed extract with the membrane of the bacterial cell, the intracellular components of the cell were released. The release of intracellular components of the cell was analyzed by measuring the A_260_ value [31, 32]. A broth culture was prepared, and test microorganisms were inoculated in the medium. The culture was then centrifuged at 10,000 rpm for 15 min, followed by washing and resuspension in 0.01 mol/L PBS solution. Different concentrations, i.e., 25, 50, 75 and 100 μg/mL of *N. sativa* seed extract and bacterial culture were mixed and incubated, and the absorbance was measured at 260 nm using a UV spectrophotometer.

### Antibiofilm assay

Biofilm formation was assessed following established protocols [33–35]. In brief, *P. aeruginosa* PAO1 overnight cultures in tryptic soy broth (TSB) were diluted in TSB to achieve an OD600 nm of 0.4. Then, 10 μL aliquots of the PAO1 suspensions with the adjusted optical density were introduced into 10 mL of fresh TSB, supplemented with *N. sativa* extract at a concentration of 7.5 μg/ml or thymoquinone at 5.5 μg/ml (Sigma Aldrich, Catalog Number: 274666-1G). The plates were incubated at 37 °C for 24 h. Following incubation, the planktonic cells were removed, and the plates underwent three consecutive washes before air-drying. The adherent bacterial cells were fixed with methanol for 25 min., followed by staining with 1% crystal violet for an additional 20 min. After removing excess dye through washing, the bound dye was dissolved in glacial acetic acid (33%), and the absorbance was measured at 590 nm using a Biotek Spectrofluorometer (Biotek, Winooski, VT, USA).

This experiment was conducted in triplicate and presented as the mean ± standard error of the mean, represented as the percentage change from the untreated control.

### Assessment of protease inhibitory effect

To assess the inhibitory impact of *N. sativa* extract and thymoquinone on protease activity, we employed the skim milk agar method, following established procedures [35, 36]. Overnight cultures of *P. aeruginosa* PAO1 grown in tryptic soy broth (TSB) containing *N. sativa* extract or thymoquinone were centrifuged at 8000 rpm for 25 min. Subsequently, 120 μL aliquots of the supernatants were dispensed into wells created in 5% skim milk agar plates. These plates were then incubated at 37 °C overnight, allowing for the measurement of clear zones that developed around the wells, which served as indicators of protease activity.

### Virtual screening

The crystal structures of *P. aeruginosa* RhlG/NADP active-site complex (PDB: 2B4Q) [37] at a resolution of 2.30 Å, the *P. aeruginosa* LasR ligand-binding domain bound to its autoinducer (PDB: 2UV0) [38] at a resolution of 1.80 Å, and the PqsR coinducer binding domain of *P. aeruginosa* with the ligand NHQ (PDB: 4JVD) [39] at a resolution of 2.95 Å were obtained from the Protein Data Bank (https://www.rcsb.org). All water molecules were removed, and hydrogen atoms were added to the crystal 3D structure of each protein with their standard geometry, followed by energy minimization.

In the preparation of the ligands, Thymol and thymoquinone were drawn using Marvin Sketch from the Marvin suite, and the lowest energy conformer for each ligand was generated. Molecular modeling simulations were carried out using the Dock module of MOE (Molecular Operating Environment), version MOE 2019.0102. The prepared thymol and thymoquinone ligands in MDL Molfile (*.mol) format were docked onto the rigid binding pocket within the active site of each of the mentioned proteins, using the flexible ligand mode. Poses for the ligand conformations were generated during the placement phase. The free binding energy of the ligand from a specific pose was calculated using the force field-based scoring function GBVI/WSA ΔG [40].

### Antibiotic susceptibility testing and synergistic effect of antibiotics and plant extract

The antibiotic susceptibility test using ampicillin (10 μg), ceftriaxone (30 μg), ciprofloxacin (5 μg) and meropenem (10 μg) was determined against the test organism using the Kirby–Bauer disk diffusion method as per CLSI 2021 guidelines. Nutrient agar media were prepared, and then the media along with petri dishes were sterilized in an autoclave at 121 °C for 45 min. After sterilization, the medium was cooled and poured into plates in a biosafety cabinet. The plates were then kept for 24 h for a sterility check. After a sterility check, a bacterial lawn was made on nutrient agar using sterile cotton swabs (Merck, Germany). Antibiotics were then applied to the culture plates and incubated at 37 °C for 24 h. Finally, the zone of inhibition was measured in mm after 24 h [41, 42].

The synergistic effect with antibiotics was determined by using the disk diffusion assay method. Nutrient agar media was prepared, and after a sterility check, a bacterial lawn was made on nutrient agar using a sterile cotton swab. Then, the bacterial lawn was prepared on petri plates. Extracts of 100 μg/mL were taken from the stock solution (1 mg/mL) and applied to antibiotic disks. The antibiotic disks were then kept in an oven at 60 °C for 10 min to be dried. Then, the disks were applied to the plates and kept in an incubator at 37 °C for 24 h. Zone of inhibition was measured in millimeters after 24 h [43–46].

### Antifungal activity

The antifungal potential of the *N. sativa* seed extract was evaluated against *Aspergillus niger, Candida albicans* and *Penicillium* [24, 47, 48]. Sabouraud dextrose agar (SDA) media was prepared and sterilized along with plates in an autoclave at 121 °C for 45 min. After performing the sterility check, the fungal species were inoculated on the plates using a sterile cotton swab and incubated at 28 °C for 5–7 days for fungal growth. Then, SDA media was prepared and sterilized along with the tubes. The tube was then cooled to 45 °C, and then 100 μL of the stock solution (2.0 mg/mL DMSO (< 1%)) of *N. sativa* extract was added to each tube. The tubes were then placed in a tilted position for slant formation. Then, fungal species were inoculated into the slant using a sterile inoculating needle. The tubes were then incubated in an incubator at 28 °C for 5–7 days. Miconazole was used as a positive control, while DMSO (< 1%) was used as a negative control. The percentage inhibition of the fungus was calculated using Eq. 1.

1 $$\left( \% \right){\bf{inhibition}} = \left( {1 - \frac{{{\bf{Linear}}\,{\bf{growth}}\,{\bf{in}}\,{\bf{test}}\,\left( {{\bf{mm}}} \right)}}{{{\bf{Linear}}\,{\bf{growth}}\,{\bf{in}}\,{\bf{control}}\,\left( {{\bf{mm}}} \right)}}} \right) \times 100$$

### Antioxidant activity

To evaluate the antioxidant activity of the prepared *N. sativa* seed extract, 2,2-diphenyl-1-picrylhydrazyl (DPPH) was used as a free radical [49–54]. In the reaction mixture, different concentrations (12.5, 25, 50, 100, 200, and 400 μg/mL) of extract were prepared for use, and the assay was performed in triplicate to decrease the uncertainty. Approximately 180 μL of DPPH solution was taken using a micropipette and mixed with 20 μL of experimental sample and then poured into each well of a titer plate. Ascorbic acid was used as a positive control, while DMSO was used as a negative control. The titer plates were then incubated at 37 °C for 30 min. The absorbance was measured at 517 nm using a microplate reader (Bio-Rad). The percent free radical scavenging assay (FRSA) was calculated using Eq. 2.

2 $$\left(\mathbf{\%}\right) \mathbf{F}\mathbf{R}\mathbf{S}\mathbf{A} =\left(1-\frac{\mathbf{A}\mathbf{b}\mathbf{s}}{\mathbf{A}\mathbf{b}\mathbf{c}} \right)\times 100$$

where Abs = absorbance of the sample and Abc = absorbance of the negative control.

### Anti-leishmanial activity

The Leishmanicidal activity of the prepared *N. sativa* seed extracts was determined against both promastigotes and amastigotes using a well-established method [55–59]. *L. tropica* KWH23 Leishmania strain was obtained from Quaid- i-Azam University Islamabad. Leishmania strains were inoculated on MI99 media supplemented with 10% FBS and then incubated. Then, 20 μL of *N. sativa* extract and 180 μL of culture solution were placed in each well of a 96-well plate. The samples were then incubated at 25 °C for 72 h. Amphotericin-B and DMSO (1%) in PBS were used as controls. After incubation, each well was filled with 20 μL of MTT (3-(4,5-dimethylthiazol-2-yl)-2,5-diphenyltetrazolium bromide) solution (4.0 mg/mL in distilled H_2_O) using a micropipette and then allowed to incubate at 25 °C for another 4 h. The absorbance was measured using a microplate reader at 540 nm. Different concentrations of the extract were prepared and employed. Equation 3 was used to calculate the percentage inhibition.

3 $$\% {\bf{Inhibition}} = \left[ {1 - \left\{ {\frac{{{\bf{Absorbance}}\,{\bf{of}}\,{\bf{sample}}}}{{{\bf{Absorbance}}\,{\bf{of}}\,{\bf{control}}}}} \right\}} \right] \times 100$$

## Results

### FTIR and GC analysis of the extract

**Fig. 2:**
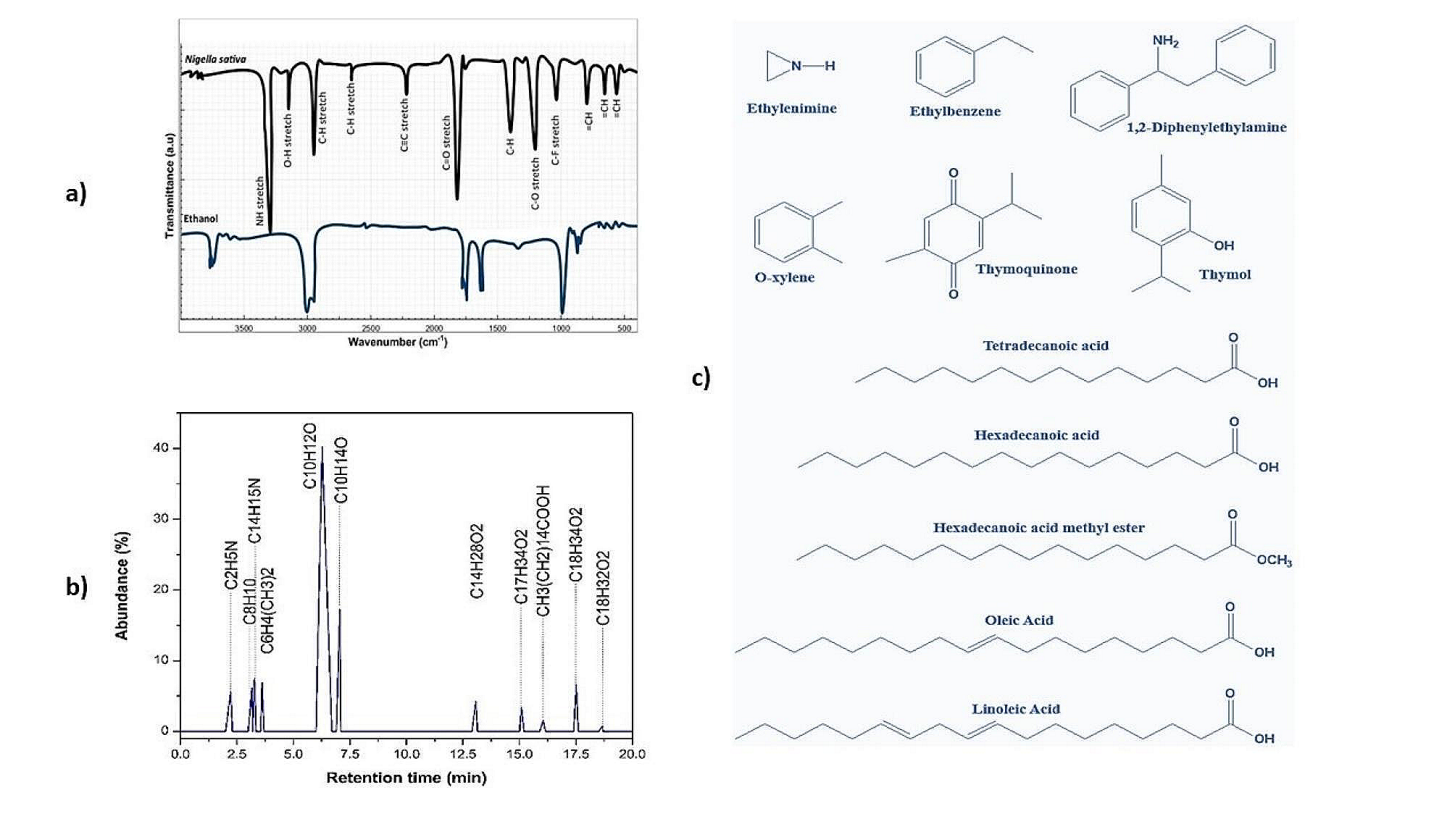
Fourier transform infrared analysis of *Nigella sativa* ethanolic extract (**a**). GC analysis and identified compounds in ethanolic extract of *N. sativa* (**b**) and chemical structure of the identified compounds (**c**)

The FTIR spectra provided valuable insights into the chemical composition, showcasing distinctive peaks corresponding to various functional groups. Notably, we observed the N-H bond stretching characteristic of amines, the C-H bond stretching associated with alkanes, the O-H bond stretching found in alcohols, the C-H stretch typical of aldehydes, the C ≡ C stretch indicative of alkynes, the C = O stretch specific to carboxylic acids, and the C-O stretch present in acids. Furthermore, we detected the C-F stretch characteristic of alkyl halides at wavenumbers of 3378, 2927, 3214, 2695, 2260, 1713, 1244, and 1046 cm^− 1^. Notably, at 918, 884, and 720 cm^− 1^, we observed a subtle alteration in the = CH bonds of alkenes, as illustrated in Fig. 2a. Employing GC, we identified 11 major compounds based on their respective peak areas and retention times (RTs), as illustrated in Fig. 2b. These compounds encompass ethylenimine, ethylbenzene, 1,2-diphenylethylamine, O-xylene, thymoquinone, thymol, and fatty acids and their esters (Fig. 2c). The spectra obtained for these compounds were compared to those of the NIST/EPA/NIH mass spectral and Wiley libraries [60, 61]. We have found that thymoquinone and thymol exist in major quantity in the extract as shown in Table 1.

**Table 1:** Compounds were identified at various retention times, along with their respective percentage compositions

Retention time	Compound	Molecular formula	Abundance/weight (%)
2.22	Ethylenimine	C_2_H_3_N	5.50
3.16	Ethylbenzene	C_8_H_10_	6.15
3.28	1,2-Diphenylethylamine	C_14_H_15_N	7.51
3.61	O-xylene	C_8_H_10_	6.90
6.27	Thymoquinone	C_10_H_12_O_2_	40.23
7.05	Thymol	C_10_H_14_O	17.23
13.07	Tetradecanoic acid	C_14_H_28_O_2_	4.20
15.09	Hexadecanoic acid methyl ester	C_17_H_34_O_2_	3.35
16.05	Hexadecanoic acid	C_16_H_32_O_2_	1.56
17.51	Oleic Acid	C_18_H_34_O_2_	6.60
18.68	9,12- Octadecadienoic acid, (z, z) (Linoleic acid)	C_18_H_32_O_2_	0.77

### Antibacterial and antifungal assay of the prepared N. sativa seed extract

**Fig. 3:**
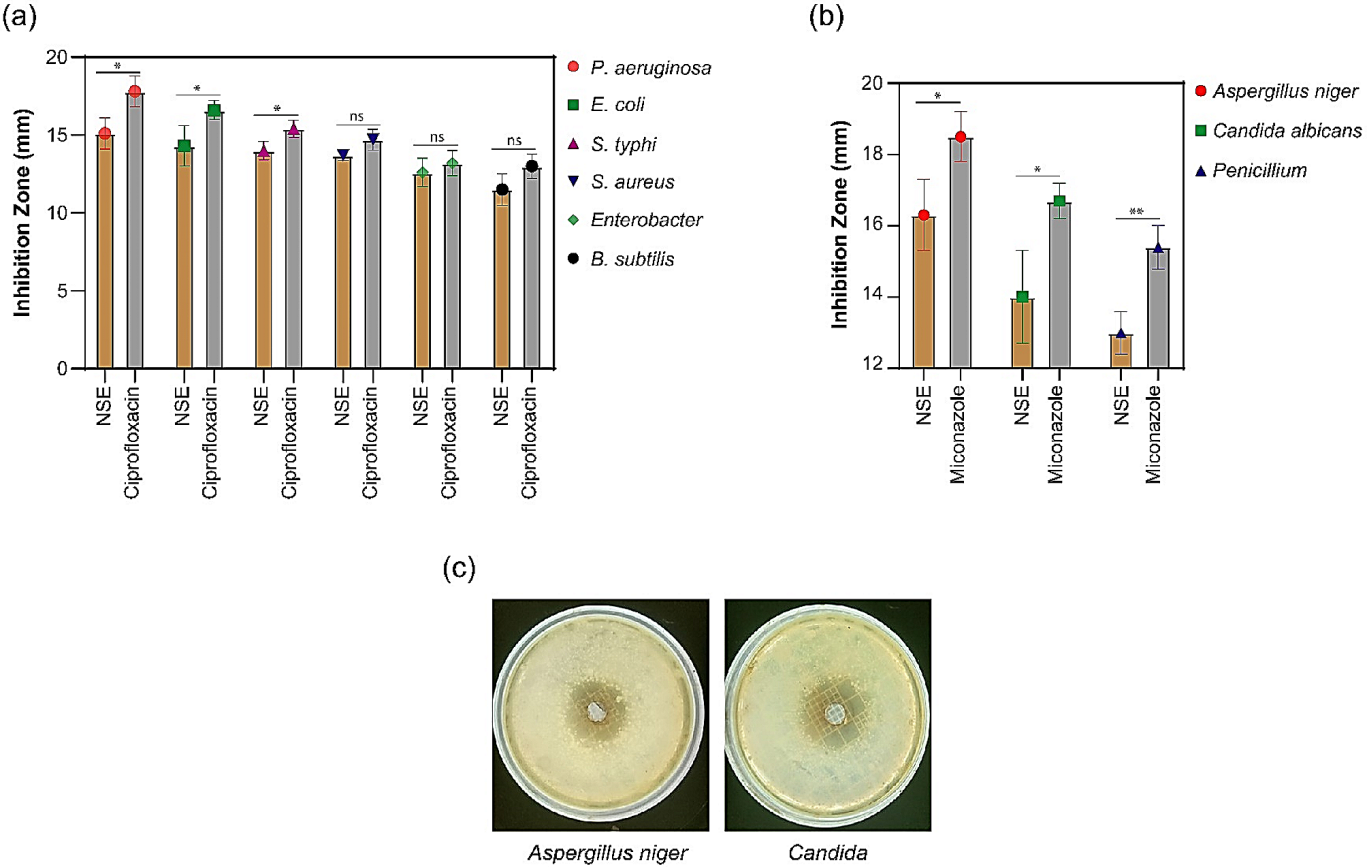
Broad spectrum antimicrobial activity of *N. sativa* extract. (**a**) antibacterial activity, (**b**) antifungal activity, and (**c**) pictures from the antifungal assay of *N. sativa* extract (NSE). Data were represented as (Mean ± SEM) of 3 independent measurements, and significance was analyzed by two-tailed unpaired T test. No significance (ns); * *p* ≤ 0.05; ** *p* ≤ 0.01

To assess the antimicrobial potential of the prepared *N. sativa* seed extract, we conducted an agar well diffusion assay. The results demonstrated notable antimicrobial activity, with the extract exhibiting the highest inhibitory zones measuring 15.1 ± 1.0, 14.3 ± 1.3, 14.0 ± 0.6, 13.7 ± 0.3, 12.6 ± 0.9, and 11.5 ± 1.0 mm against *P. aeruginosa*, *E. coli*, *S. typhi*, *Staph. aureus*, *Enterobacter*, and *B. subtilis*, respectively, as depicted in Fig. 3a. As a positive control, ciprofloxacin was employed. Furthermore, the antifungal activity of the *N. sativa* seed extract was assessed, revealing inhibitory zones measuring 16.3 ± 1.0, 14 ± 1.3, and 13.0 ± 0.6 mm against *Aspergillus niger*, *Candida*, and *Penicillium*, respectively, as illustrated in Fig. 3b. Miconazole served as the positive control in this assay. In Fig. 3c, the zones of inhibition are shown.

### Antibiotic susceptibility testing and synergistic effect of N. sativa extract

During this study, ciprofloxacin showed the highest activity (16.2 ± 0.7 mm), then ampicillin (14.4 ± 0.3 mm), ceftriaxone (14 ± 0.6 mm) and meropenem (13.2 ± 0.6 mm) against *E. coli*. Antibacterial activity against *P. aeruginosa* revealed that the maximum activity was shown by ciprofloxacin (17.1 ± 0.8 mm), then ceftriaxone (14.5 ± 0.9 mm), meropenem (14.2 ± 0.6 mm) and ampicillin (13.0 ± 0.9 mm). Against *S. aureus*, the maximum activity was shown by ceftriaxone (16.4 ± 1.0 mm), followed by ciprofloxacin (15.6 ± 0.5), meropenem (14.0 ± 1.0 mm) and ampicillin (13.6 ± 0.7 mm). The maximum activity against Enterobacter was shown by ciprofloxacin (16.1 ± 0.8 mm), followed by meropenem (15.7 ± 0.8 mm), ampicillin (13.4 ± 0.6 mm) and ceftriaxone (13.2 ± 1.0 mm). Against *Bacillus subtilis, the* highest activity was shown by ciprofloxacin (17.4 ± 0.5 mm), followed by ceftriaxone (16.1 ± 0.9 mm), meropenem (15.7 ± 0.4 mm) and ampicillin (13.2 ± 0.7 mm), while against *S. typhi*, the highest activity was shown by ciprofloxacin (16.5 ± 0.3 mm), followed by meropenem (15.2 ± 0.7 mm), ceftriaxone (14.5 ± 1.0 mm) and ampicillin (13.8 ± 0.7 mm), as summarized in Table 2.

The synergistic effect was determined using the Kirby–Bauer disk well diffusion method. Coated ciprofloxacin displayed the highest activity (19.3 ± 0.9 mm), followed by ampicillin (18 ± 0.7 mm), ceftriaxone (16.0 ± 1.0 mm) and meropenem (14.2 ± 0.6 mm) against *E. coli*. Antibacterial activity against *P. aeruginosa* revealed that the maximum activity was shown by ciprofloxacin (18.2 ± 0.6 mm), followed by ceftriaxone (17.6 ± 1.1 mm), meropenem (16.0 ± 0.3 mm) and ampicillin (15.4 ± 0.6 mm). Against *S. aureus*, the maximum activity was shown by ceftriaxone (20.1 ± 1.0 mm), followed by ciprofloxacin (18.8 ± 0.9 mm), meropenem (16.3 ± 1.2 mm) and ampicillin (15.4 ± 0.7 mm). The maximum activity against *Enterobacter* was shown by ciprofloxacin (19.2 ± 0.6 mm), followed by meropenem (17.5 ± 1.0 mm), ampicillin (16.8 ± 0.6 mm) and ceftriaxone (14.6 ± 1.0 mm). In the case of *B. subtilis*, the peak activity was shown by ciprofloxacin (20.2 ± 0.1 mm), followed by ceftriaxone (18.3 ± 0.6 mm), meropenem (16.4 ± 0.3 mm) and ampicillin (14.2 ± 0.7 mm), while against *S. typhi*, the highest activity was shown by ciprofloxacin (18.6 ± 0.4 mm), followed by meropenem (17.9 ± 0.8 mm), ceftriaxone (17.2 ± 1.0 mm) and ampicillin (15.5 ± 0.9 mm), as shown in Table 2. The increase in potency was calculated by using Eqs. 4 and 5.

4 $$Inhibition \left(\%\right)=\frac{{ZI}_{observed}}{{ZI}_{CLSI}}\times 100$$

5 $$Potency \left(\%\right)= \frac{{ZI}_{Extrcat-coated}-{ZI}_{non-coated }}{{ZI}_{CLSI}}\times 100$$

**Table 2:** Antibacterial activity of noncoated and *N. sativa* seed extract-coated antibiotics and their synergistic effect

Test Organisms	Antibiotics	CLSI Standard (mm)	Zone of Inhibition of Non-Coated antibiotics (mm)*	Zone of Inhibition of Coated antibiotics (mm)*	Increase potency (%)
*P. aeruginosa*	Ciprofloxacin	21	17.1 ± 0.8	18.2 ± 0.6	5.3 ^ns^
	Ampicillin	15	13.0 ± 0.9	15.4 ± 0.6	**16.0** ^*^
	Ceftriaxone	21	14.5 ± 0.9	17.6 ± 1.1	14.8 ^ns^
	Meropenem	18	14.2 ± 0.6	16.0 ± 0.3	**10.0** ^**^
*E. coli*	Ciprofloxacin	21	16.2 ± 0.7	19.3 ± 0.9	**14.8** ^**^
	Ampicillin	15	14.4 ± 0.3	18 ± 0.7	**24.0** ^**^
	Ceftriaxone	21	14 ± 0.6	16.0 ± 1.0	**9.4** ^**^
	Meropenem	18	13.2 ± 0.6	14.2 ± 0.6	5.6 ^ns^
*S. typhi*	Ciprofloxacin	21	16.5 ± 0.3	18.6 ± 0.4	**10.0** ^**^
	Ampicillin	15	13.8 ± 0.7	15.5 ± 0.9	11.3 ^ns^
	Ceftriaxone	21	14.5 ± 1.0	17.2 ± 1.0	**12.9** ^**^
	Meropenem	18	15.2 ± 0.7	17.9 ± 0.8	**15.0** ^*^
*S. aureus*	Ciprofloxacin	21	15.6 ± 0.5	18.8 ± 0.9	**15.2** ^**^
	Ampicillin	15	13.6 ± 0.7	15.4 ± 0.7	**12.0** ^*^
	Ceftriaxone	21	16.4 ± 1.0	20.1 ± 1.0	**25.7** ^*^
	Meropenem	18	14.0 ± 1.0	16.3 ± 1.2	13.1 ^ns^
*Enterobacter*	Ciprofloxacin	21	16.1 ± 0.8	19.2 ± 0.6	**14.7** ^**^
	Ampicillin	15	13.4 ± 0.6	16.8 ± 0.6	**22.7** ^**^
	Ceftriaxone	21	13.2 ± 1.0	14.6 ± 1.0	6.6 ^ns^
	Meropenem	18	15.7 ± 0.8	17.5 ± 1.0	10.0 ^ns^
*B. subtilis*	Ciprofloxacin	21	17.4 ± 0.5	20.2 ± 0.1	**13.3** ^***^
	Ampicillin	15	13.2 ± 0.7	14.2 ± 0.7	6.7 ^ns^
	Ceftriaxone	21	16.1 ± 0.9	18.3 ± 0.6	**10.4** ^*^
	Meropenem	18	15.7 ± 0.4	16.4 ± 0.3	3.9 ^ns^

Data were represented as (Mean ± SEM) of 3 independent measurements, and significance was analyzed by two-tailed unpaired T test. Bold numbers indicate significance, no significance (ns); * *p* ≤ 0.05; ** *p* ≤ 0.01; *** *p* ≤ 0.001

### Membrane damage assay

*N. sativa* seed extract was employed against various bacterial strains, including *P. aeruginosa*, *Enterobacter*, *Staph. aureus*, *E. coli*, *S. typhi*, and *B. subtilis*. The absorbance at 260 nm (A260) was monitored over time. After exposure to *N. sativa* seed extract for 60 min., the A260 values were recorded as follows: 35.14 ± 0.13 for *P. aeruginosa*, 58.14 ± 0.29 for *E. coli*, 88.32 ± 0.13 for *S. typhi*, 68.3 ± 0.52 for *S. aureus*, 83.47 ± 0.52 for *Enterobacter*, and 28.56 ± 0.45 for *B. subtilis*, all at a concentration of 50 μg/mL, as detailed in Table 3. This observed activity can be attributed to the extract’s ability to penetrate bacterial cells, leading to membrane disruption and subsequent expulsion of internal cellular materials.

**Table 3:** Membrane Damage Assay of *N. sativa* seed extract

Bacterial species	P. aeruginosa	E. coli	S. typhi	S. aureus	Enterobacter	B. subtilis
% Damage	35.14 ± 0.13	58.14 ± 0.29	88.32 ± 0.13	68.3 ± 0.52	83.47 ± 0.52	28.56 ± 0.45

### Minimum inhibitory concentration (MIC) and bacterial antibiofilm and antiprotease activity

The minimum inhibitory concentration was determined by using the serial dilution method in a 96-well microtiter plate. *E. coli* showed inhibition at 10 μg/mL, while *S. aureus*, *Enterobacter*, and *S. typhi* showed inhibition at 20 μg/mL and *P. aeruginosa*, and *B. subtilis* showed inhibition at 30 μg/mL taken from the stock solution (1.0 mg/mL). Similarly, we measured the MIC of thymoquinone (the major active constituent of *N. sativa* extract). TQ showed remarkable antibacterial activity with MICs comparable to those of *N. sativa* extract against the same group of microbes, as shown in Table 4.

**Table 4:** Minimum inhibitory concentration of *N. sativa* seed extract and thymoquinone (TQ)

Bacterial species	P. aeruginosa	E. coli	S. typhi	S. aureus	Enterobacter	B. subtilis
MIC (Extract)	30 μg/ml	10 μg/ml	20 μg/ml	20 μg/ml	20 μg/ml	30 μg/ml
MIC (TQ)	22 μg/ml	7 μg/ml	14 μg/ml	16 μg/ml	15 μg/ml	24 μg/ml

**Fig. 4:**
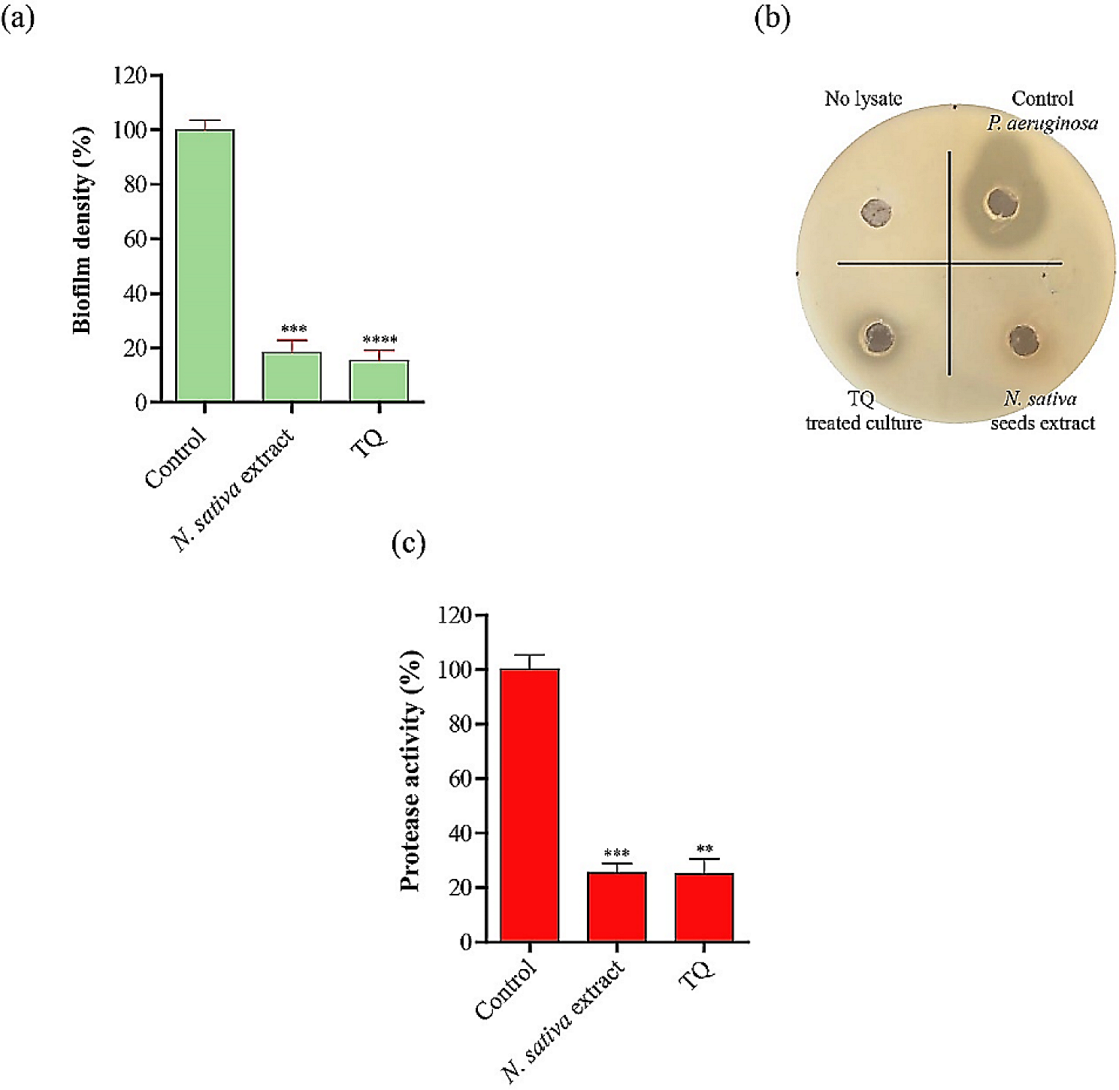
N. sativa and thymoquinone disrupt the bacterial biofilm and compromise the protease activity of *P. aeruginosa*. **a**) Effect of *N. sativa* and thymoquinone on *P. aeruginosa* biofilm formation and (**b** & **c**) protease inhibition using the skim milk agar method. Two hundred microliters of supernatant from different cultures with *N. sativa* or thymoquinone was tested for protease activity on skim milk agar by a well diffusion assay. Data are presented as the mean ± standard error of the mean (SEM). ** *p* ≤ 0.01; *** *p* ≤ 0.001, **** *p* ≤ 0.0001. A two-tailed unpaired T test was employed to analyze significance

Next, we investigated the ant virulence activity of *N. sativa* seed extract or pure thymoquinone against *P. aeruginosa*. We examined their ability to disrupt the formation of bacterial biofilms and proteases, which are crucial virulence factors for pathogenesis and spreading *P. aeruginosa* infection, especially in cases of wound and burn infections. A sub inhibitory concentration (1/8th MIC) of *N. sativa* extract or thymoquinone (TQ) remarkably inhibited the formation of biofilms (∼ 82% reduction in the biofilm density in case of *N. sativa* extract and ∼ 85% in case of TQ) and blocked the protease activity (∼ 75% reduction in the protease activity in both cases) of treated *P. aeruginosa* cultures relative to cultures that were not treated with *N. sativa* extract or TQ (Fig. 4a, b, and **c**).

### Virtual screening and molecular docking

**Fig. 5:**
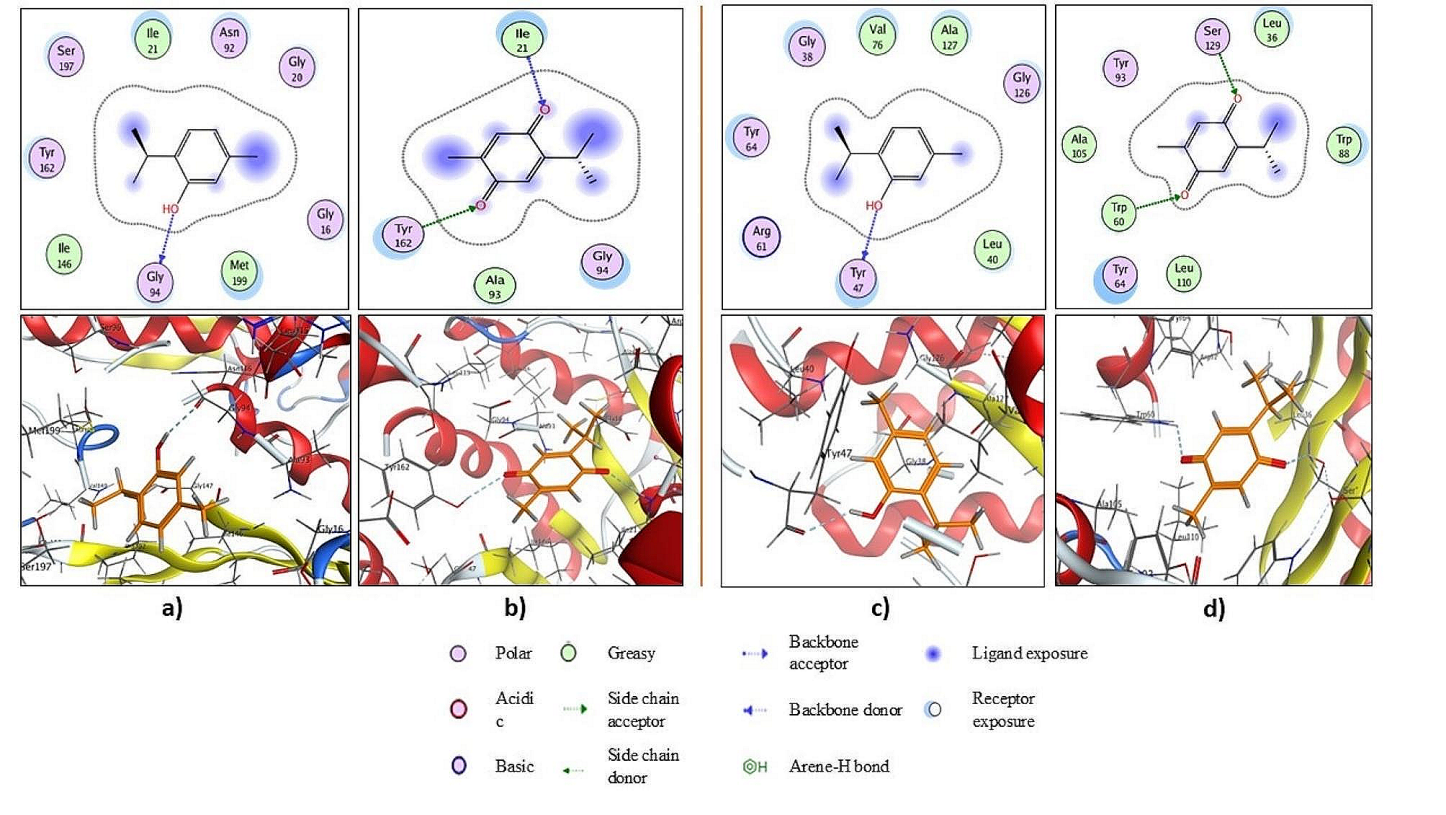
2D and 3D interactions of thymol (**a**) and thymoquinone (**b**) with the binding site of the crystal structure of the *P. aeruginosa* RhlG/NADP active-site complex (PDB: 2B4Q)2D and 3D interactions of thymol (**c**) and thymoquinone (**d**) with the binding site of the crystal structure of the *P. aeruginosa* LasR ligand-binding domain bound to its autoinducer (PDB: 2UV0)

Molecular modeling simulation as a computational technique was carried out to understand the binding efficacy of a certain ligand to its respective macromolecular target. Docking results (Fig. 5a) of thymol onto the macromolecule *P. aeruginosa* RhlG/NADP active-site complex (PDB: 2B4Q) revealed that a conspicuous H-bond was constructed between the hydrogen atom of the hydroxyl group and the H-acceptor backbone of the conserved amino acid Gly94. Moreover, the hydrophobic/hydrophilic interactions revealed from the cyan-shaded amino acids Gly16, Gly20, Ile21, Asn92, Gly94, Ile146, Tyr162 and Ser197 at the receptor side and blue-shaded moieties at the ligand side enhanced the overall recognition and augmented the ligand/receptor complex to achieve free binding energy of 9.91211796 kcal/mol. However, (1,4- or p-) quinone-based thymoquinone formed two H-bonds; the first H-bond was built up between the *sp2* hybridized oxygen at position − 1, which acted as an H-bond acceptor, and the H-bond donor backbone of the conserved amino acid Ile21, whereas the second H-bond was formed between the *sp2* hybridized oxygen at position − 4 and the H-bond donor side chain of the conserved amino acid Tyr162 as shown in Fig. 5b. Thus, the thymoquinone ligand scored a free energy of binding of -8.68101788 Kcal/mol.

Undoubtedly, the aforementioned conserved amino acids Gly16, Gly20, Ile21, Asn92, Gly94, and Tyr162 are involved in the interactions with the internal autoinducer [1]. 

On the other hand, (Fig. 5c), we docked thymol against the crystal structure of the *P. aeruginosa* LasR ligand-binding domain bound to its autoinducer (PDB: 2UV0), and we found that the active functional hydroxyl group via its hydrogen formed an H-bond with the H-bond acceptor backbone of the conserved amino acid Tyr47. In addition, for the hydrophobic/hydrophilic interactions, the ligand/receptor complex achieved a free energy of binding of -10.0491753 kcal/mol. While thymoquinone formed two H-bonds via its p-quinoidal base as shown in Fig. 5d, the *sp2* hybridized oxygen at position − 1 accepted a H-bond from the side chain of the conserved amino acid Ser129, whereas the *sp2* hybridized oxygen at position − 4 accepted an H-bond from the conserved amino acid Trp60, giving rise to a score-free binding energy of -10.0251846 kcal/mol.

Reportedly, the conserved amino acid Tyr47 was involved in hydrophobic/ hydrophilic interactions with the natural ligand whereas Trp60 and Ser129 were involved in formation of H-bond interactions with it [2]. 

**Fig. 6:**
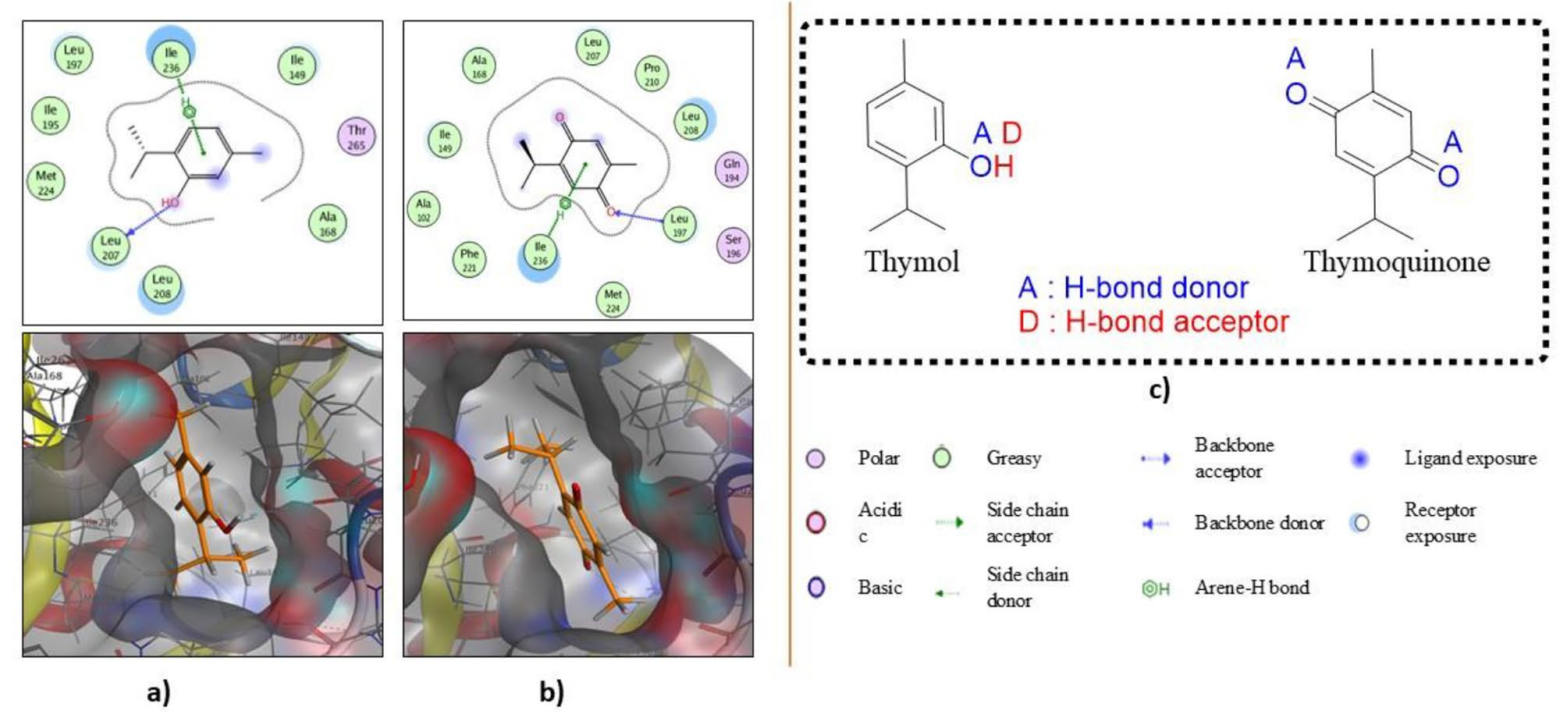
2D and 3D interactions of thymol (**a**) and thymoquinone (**b**) with the binding site of the crystal structure of the *PqsR* coinducer binding domain of *P. aeruginosa* with the ligand NHQ (PDB: 4JVD). The structures of thymol and thymoquinone (**c**)

However, docking of thymol onto the crystal structure of the PqsR coinducer binding domain of *P. aeruginosa* with the ligand NHQ (PDB: 4JVD) showed that the aromatic ring in thymol acted as a nonclassical Lewis base and formed arene − H with the conserved amino acid Ile236 as shown in Fig. 6a. Additionally, an H-bond that was formed between the hydrogen of the hydroxyl group and the backbone of the conserved amino acid Leu207 enhanced the ligand/receptor complex stability to achieve a free energy of binding of 10.1676149 kcal/mol. Likewise, the quinoidal moiety in thymoquinone formed an arene − H bond with Ile236, and an H-bond was constructed between the *sp2* hybridized oxygen at position − 4 and the amino acid Leu197, resulting in a total free binding energy score of -8.45744038 kcal/mol (Fig. 6b).

However, the conserved amino acids Leu207, Ile236 were reported to be involved in strong hydrophobic/ hydrophilic interactions with the autoinducer [3]. 

Of note, both thymol and thymoquinone exhibited their best binding profiles with binding sites in the crystal structures of the PqsR coinducer binding domain of *P. aeruginosa* with the ligand NHQ and the *P. aeruginosa* LasR ligand-binding domain bound to its autoinducer, respectively. Moreover, thymol appeared dominant over thymoquinone throughout the molecular simulation study; undoubtedly, this is ascribed to the phenolic characteristics of thymol, which possesses H-bond donor and acceptor sites (Fig. 6c), in addition to the higher density of electron clouds inside its ring, which normally raises the ability to bond as a nonclassical base. Thymol is worthy of further investigation toward the discovery of biofilm formation inhibitors in *P. aeruginosa* as drug candidates.

### Antioxidant activity and anti-leishmanial activity

**Fig. 7:**
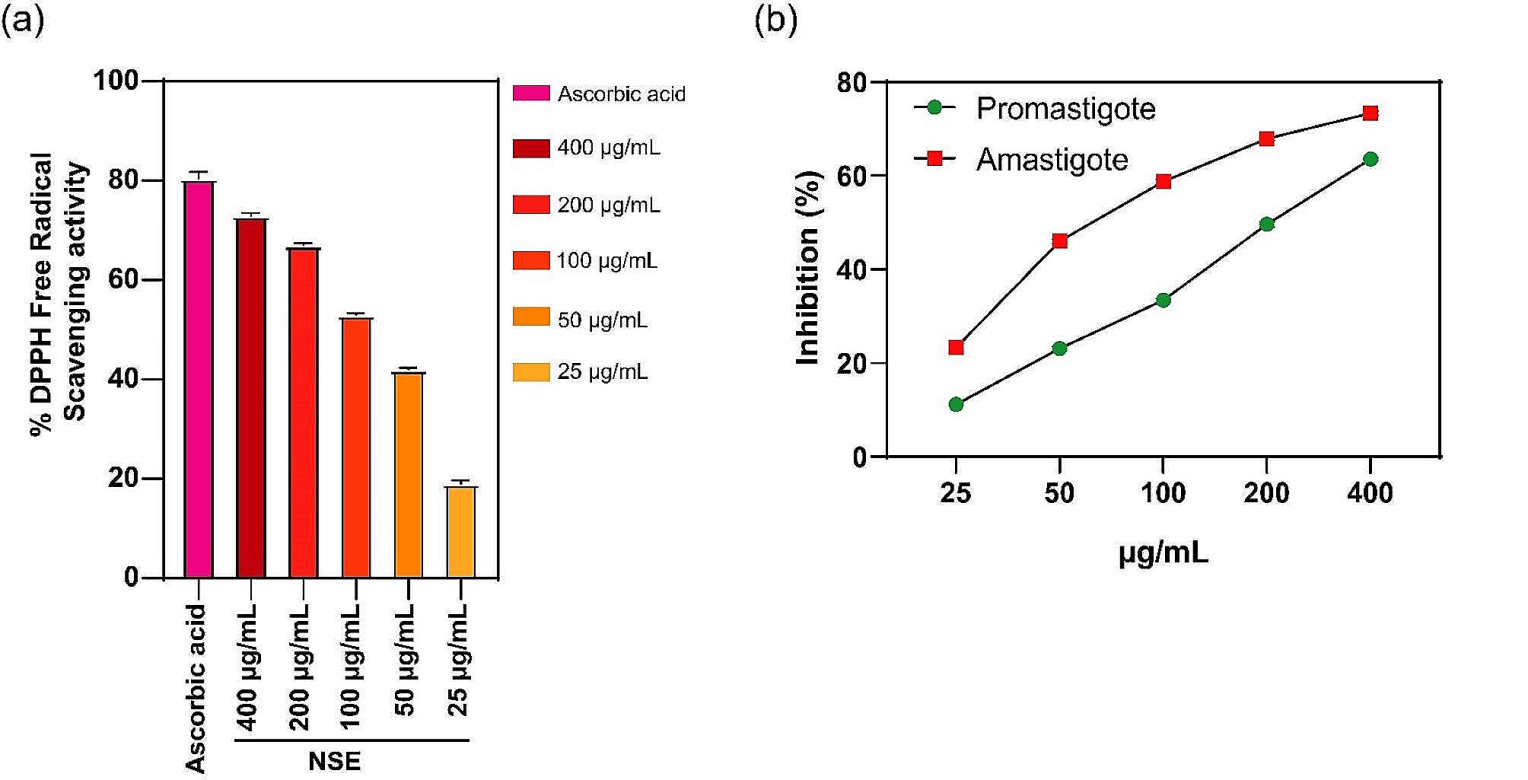
(**a**) antioxidant activity and (**b**) anti-leishmanial activity of *N. sativa* extract (NSE)

The antioxidant activity of *N. sativa* seed extract was assessed using the DPPH free radical scavenging assay. The extract demonstrated noteworthy DPPH free radical scavenging activity, surpassing that of the standard, ascorbic acid. Various concentrations of *N. sativa* seed extract (400, 200, 100, 50, and 25 μg/mL) were tested. The highest DPPH activity, measuring 73.1 ± 0.21%, was observed at the highest concentration of 400 μg/mL, while the activity decreased to 19.221 ± 0.31% at the lowest concentration of 25 μg/mL. Ascorbic acid was employed as a reference control. These activities at different concentrations are presented in Fig. 7a.

Leishmania, a genus of trypanosomes transmitted by sandfly vectors, causes leishmaniasis. Amastigote forms of the parasite reside in human mononuclear phagocytes and the circulatory system, while promastigote forms inhabit the digestive tracts of sandflies. This study evaluated the effect of *N. sativa* seed extract at concentrations of 25, 50, 100, 200, and 400 μg/mL on promastigote and amastigote cultures of *L. tropica* using the MTT assay. At the highest concentration of 400 μg/mL, the cytotoxicity displayed a dose-dependent pattern, resulting in significant mortality rates of 63.54 ± 0.26% and 73.36 ± 0.47% for the promastigote and amastigote forms of the parasite, respectively, as illustrated in Fig. 7b.

## Discussion

The exploration of novel antimicrobial agents becomes imperative due to the escalating prevalence of antibiotic-resistant bacteria and the need for alternative treatment modalities [16, 62]. Medicinal plants, along with their active constituents, exhibit substantial antimicrobial efficacy, positioning them as promising candidates against resistant microbes. Integrating medicinal plants into clinical practices not only expands therapeutic options but also underscores the importance of tapping into nature’s pharmacopeia in the ongoing battle against infectious diseases. Ongoing research and development hold promise for discovering new plant-based medicines, offering sustainable and effective solutions to global health challenges [63–65].

Notably, *N. sativa* seed extracts exhibit remarkable therapeutic potential across a spectrum of diseases, including bacterial infections, arthritis, cancer, hypertension, ulcers, and epilepsy [10]. Previous studies have substantiated the antimicrobial capabilities of *N. sativa* extract [66–68]. According to Duke’s ethnobotanical and phytochemical database [69], thymol, thymoquinone, and other compounds collectively contribute to its antimicrobial, antioxidant, anti-inflammatory, and anticancer activities.

In our study, we prepared an ethanolic extract of *N. sativa* seeds and comprehensively assessed its phytochemical and antimicrobial properties. Our findings revealed the highest antibacterial activity against gram negative bacteria followed by the gram positive, this aligns with similar studies highlighting *N. sativa*’s effectiveness against various bacteria and fungi [67–76]. The antimicrobial activity is attributed to the interaction between phytochemicals and the bacterial cell membrane, leading to the release of intracellular macromolecules. Notably, the extract efficiently disrupted the formation of the bacterial extracellular matrix (biofilm) and inhibited protease activity, suggesting potential activity in attenuating bacterial virulence factors [35].

Virtual screening affirmed the unique affinity of thymol and thymoquinone (the major constituents of the extract) to bind and compromise key regulatory proteins of biofilm formation.

The remarkable efficacy of black seed extract in disrupting bacterial membranes and removing bacterial biofilm further reinforces the penetration of antibiotics, such as ciprofloxacin and beta-lactams, synergizing their bactericidal action.

*N. sativa* seed extract demonstrated significant antifungal activity against both yeast and filamentous fungi in accordance to previous studies [73]. Additionally, according to our study and others the extract exhibited substantial inhibitory effects against various parasite forms [77–79], displaying significant cytotoxicity against both promastigote and amastigote forms, attributed to oxidative stress induction and inactivation of cellular proteins [78]. Finally, *N. sativa seed* extract exhibited noteworthy antioxidant potential in the DPPH assay [54], especially at a concentration of 400 μg/mL, providing additional benefits in mitigating cellular damage.

In conclusion, our study contributes and confirms valuable insights into the therapeutic potential of *N. sativa*, potentially paving the way for the development of novel natural medicines or supplements with potent antibacterial and antioxidant properties [80, 81].

## Conclusions

The study on N. sativa seed extract revealed its broad antimicrobial effectiveness against various bacterial and fungal strains. Notably, combining the extract with coated antibiotics, particularly ciprofloxacin, demonstrated promising synergistic antibacterial effects. The extract induced bacterial membrane damage and exhibited significant antioxidant activity. Furthermore, it displayed dose-dependent cytotoxicity in anti-leishmanial activity against *Leishmania tropica*. These findings advance our comprehension of *N. sativa*’s therapeutic potential, paving the way for future exploration into natural remedies or supplements with potent antibacterial and antioxidant properties.

## Data Availability

The datasets used and/or analysed during the current study can be obtained from the corresponding author upon reasonable request.
